# Microbiological quality of raw drinking milk and unpasteurised dairy products: results from England 2013–2019

**DOI:** 10.1017/S0950268820001016

**Published:** 2020-05-14

**Authors:** J. McLauchlin, H. Aird, A. Elliott, E. Forester, F. Jørgensen, C. Willis

**Affiliations:** 1 Public Health England, National Infection Service, Food Water and Environmental Microbiology Services, Colindale, London, UK; 2 Public Health England, National Infection Service, Food Water and Environmental Microbiology Laboratory York, National Agri-Food Innovation Campus, York, UK; 3 Public Health England, National Infection Service, Food Water and Environmental Microbiology Laboratory Porton, Porton Down, Salisbury, UK; 4 Public Health England, National Infection Service, Field Services, South West, Bristol, UK; 5 Public Health England, National Infection Service, Field Services, North West Office, Liverpool, UK

**Keywords:** Foodborne infection, microbiological quality, raw drinking milk, unpasteurised dairy products

## Abstract

The aim of this study was to review microbiology results from testing >2500 raw drinking milk and dairy products made with unpasteurised milk examined in England between 2013 and 2019. Samples were collected as part of incidents of contamination, investigation of infections or as part of routine monitoring and were tested using standard methods for a range of both pathogens and hygiene indicators. Results from testing samples of raw cow's milk or cheese made from unpasteurised milk for routine monitoring purposes were overall of better microbiological quality than those collected during incident or investigations of infections. Results from routine monitoring were satisfactory for 62% of milks, 82% of cream, 100% of ice-cream, 51% of butter, 63% of kefir and 79% of cheeses, with 5% of all samples being considered potentially hazardous. Analysis of data from cheese demonstrated a significant association between increasing levels of indicator *Escherichia coli* with elevated levels of coagulase positive staphylococci and decreased probability of isolation of Shiga toxin-producing *E. coli*. These data highlight the public health risk associated with these products and provide further justification for controls applied to raw drinking milk and dairy products made with unpasteurised milk.

## Introduction

There has been an increased consumer demand for drinking raw milk [1, 2], and dairy products made from unpasteurised milk (including raw and thermised milk) such as cheese, butter, cream, ice-cream and kefir. There has also been an increase in the popularity of consuming raw milk and associated products produced from non-bovine species such as goat, sheep, horse, donkey and camel [3, 4].

The consumption of raw milk and unpasteurised dairy products can present health risks from contamination by a variety of pathogenic micro-organisms, with more risks being reported in association with cows' milk as compared to other species such as goats and sheep [5, 6]. However, there is comparatively little current data available for other unpasteurised dairy products consumed in the UK [7–10].

Food on sale in England is regulated as part of the Official Controls [11] and, because raw cow's milk has been categorised as a ‘risky food’, there are restrictions for the sale of cow's milk for drinking [1, 12]. Raw cow's milk for drinking can only be sold at farms and farm shops at the point of production, which is also extended to local deliveries and farmer's markets. Sale is prohibited at town markets, village fetes, school fetes, pop concerts, horse events, car boot sales, agricultural shows or laybys on the side of a road [12]. These restrictions do not apply to milk from other species or other dairy products made from unpasteurised milk.

The availability of published microbiological criteria for the interpretation of results in these products is variable. European Regulation EC 2073/2005 (as amended [13]) sets limits for *Listeria monocytogenes* in all ready-to-eat foods, as well as *Escherichia coli* levels in cream and butter made from unpasteurised milk, and coagulase positive staphylococci (CPS) in cheese made from unpasteurised milk. Raw drinking milk is not covered in this regulation but criteria for hygiene indicator tests (coliforms and aerobic colony count (ACC)) are specified in English legislation (Food Hygiene [England] Regulations, 2013). The Health Protection Agency Guidelines for ready-to-eat foods [14] indicate the need for pathogen and indicator organism testing and provide interpretations for a range of bacterial species. The Specialist Cheesemakers have also provided interpretations for *E. coli*, Enterobacteriaceae and Shiga toxin producing *E. coli* (STEC) in raw cheeses for manufacturers (http://www.specialistcheesemakers.co.uk/). Guidance and microbiological criteria are also available for local authority for raw cheese enforcement from the Scottish Food Enforcement Group, particularly for the control of STEC (https://www.foodstandards.gov.scot/downloads/Guidance_for_Local_Authorities_–_Cheese_made_from_Unpasteurised_Milk_-_May_2019.pdf).

Public Health England (PHE) manages a network of Food Water and Environmental Microbiology (FW&E) Laboratories which routinely examines dairy products for the presence of a range of bacterial pathogens and indicator organisms. Food samples are collected by Environmental Health Practitioners as part of their responsibility to enforce food safety legislation, monitoring food business operators or investigating cases of potential foodborne illness. Samples are also submitted to the PHE FW&E laboratories by producers who wish to perform their own verification checks on their products. In 2013, the FW&E laboratory network invested in and implemented a single laboratory information management system (LIMS). By mid-2019, at the time of writing (August 2019) this LIMS had microbiological results and associated data on more than 240 000 food samples. This database represents a resource for outbreak investigation, as well as for hazard analysis, risk assessment, risk management and risk communication [6, 15]. The aim of this study was to review results held within this LIMS which were obtained from testing both raw drinking milk and dairy products made with raw or thermised milk examined between 2013 and 2019.

## Materials and methods

### Sample collection

Data on the testing of all samples of raw drinking milk and other unpasteurised milk products tested during 2013–2019 were extracted from the FW&E LIMS. This dataset included samples collected from the point of sale as well as the point of manufacture and were sampled for routine monitoring, in response to hygiene concerns as well as part of outbreak investigations. Results of testing of finished product either at the point of production, on retail sale or in catering environments were included: product collected during maturation or food ingredients (e.g. raw milk used for cheese making or cheese curds) were not included in this analysis. In some instances, routine monitoring was carried out at production sites associated with incidents, but these were only considered as ‘routine’ following at least two sets of satisfactory clearance samples and at least 1 month after the completion of the incident investigation. Results on testing raw bovine milk for drinking that had been examined between 2014 and 2016 were excluded since these have been published previously [6].

Samples were collected in England and transported in accordance with the Food Standards Agency Food Law Practice Guidance [16] and examined in one of the network of PHE Official Food Control Laboratories located in Birmingham, London, Preston, Porton and York.

Data were collected on each individual sample which, together with the original data collected at the time of sampling, was annotated by internet searches. Cheeses made from raw or thermised milk were classified by type (hard, semi hard, semi soft, soft, blue and fresh) according to the Codex General Standards for cheese [17]. Additional data on cheese type, ingredient milk species and the range of products sold by individual dairies were added following manual searches of manufacturer's web sites as well as data from the Alphabetical List of Cheese (https://cheese.com/alphabetical/), the Specialist Cheese Makers Association (http://www.specialistcheesemakers.co.uk/) and the FSA's register of Raw Drinking Milk Premises in England, Wales and Northern Ireland (https://data.gov.uk/dataset/f6706084-9c82-4a50-a781-41e0e6229948/raw-drinking-milk-premises-in-england-wales-and-northern-ireland, 1st August 2019 update).

Results from testing a total of 2529 samples of raw milk and products made from unpasteurised milk and collected in England between February 2013 and April 2019 were identified in the PHE LIMS database. This dataset included: 719 raw cow's milks (2017–2019), 584 raw milks from non-bovine animals (2013–2019); 100 cream, two ice-cream, 37 butter, 24 kefir and 1063 cheeses (all from 2013–2019). The mean annual total was 360 samples examined and varied between 823 tested in 2017 and 101 in 2019 (January to April only).

The characteristics of the 2529 samples and the sampling settings for each of the various dairy products is shown in Table 1. Amongst all the samples, 56 bovine milks and 79 cow or goats milk cheeses were associated with six incidents or outbreaks of infection which are summarised in Table 2.

**Table 1.** Characteristics of the 2529 samples and sampling settings for raw milk and dairy products made from unpasteurised milk

Types of dairy products made from unpasteurised milk (sampling period)	Number tested	Sampling settings
Cow's drinking milk (2017–2019)	719	Fifty-six samples collected from three separate incidents of infection. The remaining 663 samples were routine monitoring of 126 dairies with between 1 and 39 samples tested per dairy.
Non-bovine drinking milks: 534 were from goats, 15 from sheep, 28 from buffalo and 7 from camel (2013–2019)	584	No samples in association with incidents or infections. All samples were collected as part of routine monitoring, 518 were from the point of production (58 dairies with between one and 65 samples tested per dairy). Ten samples were collected from retail sale and the sampling location for the remaining five could not be established. The 15 raw sheep's milk samples were all collected from four dairies at the point of production. Twenty seven of the 28 buffalo milks were from five dairies at production, one was from retail. The 7 camel milks were all collected at retail.
Cream prepared from cow's milk: 98 described as double cream, one as crème fraiche, and one as cream (2013–2019)	100	Two samples came from a producer that was also associated with a STEC outbreak linked to raw milk consumption [18, 19]. All other samples were tested for routine monitoring purposes. Three samples were collected from shops and a market, the remaining 97 samples were sampled from 11 different producers, all of which also sold raw cow's milk for drinking.
Ice-cream prepared from goat's milk (2013–2019)	2	Not associated with incidents or infections. Both samples were tested for routine monitoring purposes and collected at the point of production.
Butter prepared from cow's milk (2013–2019)	37	No samples submitted in association with incidents or infections. All samples were tested for routine monitoring purposes. Thirty-five samples were collected from the point of production from nine different dairies, all of which also reported selling raw cow's milk for drinking. Two samples were from retail premises (a farm shop and a market).
Kefir prepared from goat's milk (18 samples) or cow's milk (six samples) (2013–2019)	24	No samples submitted in association with incidents. All samples were tested for routine monitoring purposes and were sampled at the point of production from eight different premises (16 were sampled from the same premises), all of which also sold raw drinking milk
Cheese prepared from cow's milk (769 samples), goat's milk (178 samples), sheep's milk (94 samples), buffalo or cow and buffalo milk (22 samples) (2013–2019)	1063	Seventy-nine samples (35 prepared from cow's milk and 44 from goat's milk) were collected from three incidents or outbreaks of infection. All 984 remaining samples were tested for routine monitoring purposes: 814 (85%) were collected from the point of production (either from the production environment or on sale at the dairies), 126 (13%) at retail and 11 (1%) from catering establishments (hotels and restaurants). The settings for the remaining 11 (1%) could not be established. The cow, sheep and goat's milk cheeses sampled at the point of production were made at 98 dairies (between 1 and 94 samples tested per dairy) and of these 12 dairies (12%) were also registered to produce raw milk for drinking.

**Table 2.** Summary of microbiological results of testing samples of raw milk and unpasteurised milk cheeses associated with incidents and outbreaks of infection

Incident or outbreak of infection number [references]	Description	Incriminated food	Samples collected	Results of microbiological analysis
Milk outbreak 1 [19, 20]	Seven cases of STEC O157:H7 PT 21/28 *stx2* in 2017	Consumption of raw cow's milk from a single dairy	21 samples of raw cow's milk and two samples of cream collected from the dairy	STEC O157:H7 indistinguishable from that isolated from the cases and from cow faeces collected on the farm was isolated from three bulk tank milk samples. Statutory indicator bacteria tests (ACCs and coliforms) gave compliant results for all three samples from which STEC was isolated: borderline levels of CPS were detected in all three samples. Of the remaining 18 samples, 13 had borderline levels of CPS, three had unsatisfactory levels of coliforms and three had unsatisfactory levels of ACCs. None of the 21 samples were interpreted as being of satisfactory quality. The two samples of cream were of satisfactory quality and were tested for *E. coli*, *Listeria* and *Salmonella*.
Milk outbreak 2 [19]	Four cases of *Campylobacter* infection in 2017	Consumption of raw cow's drinking milk from a single dairy	31 samples of raw cow's milk collected from the dairy	*Campylobacter jejuni* MLST 22 was isolated from two milk samples which were indistinguishable from that infecting the patients: results for CPS were borderline for one sample and the level of coliforms was unsatisfactory for the other; all other microbiological parameters (ACC, *L. monocytogenes*, *Salmonella* and STEC) were satisfactory. For the remaining 29 samples, 12 were satisfactory for all parameters, four had borderline levels of CPS and 13 had unsatisfactory levels of coliforms.
Milk outbreak 3 [19]	Single sporadic case of *S. enterica* serovar Dublin in 2017	Consumption of raw cow's drinking milk at a childminders which was bought from a local on farm dairy	Four samples of raw cow's milk from the bulk tank and a filter sock removed following milking at the dairy	*S*. Dublin was detected in all the milk samples (as well as a filter sock) and isolates were found to be indistinguishable from the clinical isolate. All four milk samples from which *S*. Dublin was recovered were satisfactory for all other parameters (ACC, coliforms, *Campylobacter*, *L. monocytogenes*, CPS)
Cheese collected following an STEC outbreak associated with raw drinking milk consumption also from these premises [19, 21]	Sporadic case of *S. enterica* serovar Mbandaka (MLST: 413). STEC was also isolated from this patient, in 2014	This cheese was manufactured at the same farm that had previously been associated with an STEC outbreak	Seven samples of cow's milk cheese collected at the farm cheese manufacturing environment in 2015 which was co-located with the dairy producing the milk	*S.* Mbandaka indistinguishable from the patient was isolated from one cheese sample. Of all seven samples, *Listeria*, CPS, STEC and *E. coli* O157 were not detected. Two samples were satisfactory with respect to the presence of *E. coli*, one was borderline, and the remaining four (including the sample from which *S.* Mbandaka was isolated) were unsatisfactory, with results ranging from 10^2^ to 10^4^ cfu/g.
Cheese infection [19]	Sporadic case of listeriosis infected with *L. monocytogenes* serovar 1/2a, ST403 and SNP type 1.1.1.1.1.1.1 in 2016	The patient purchased the cheese from a farm shop which was co-located with the cheese production environment	28 samples of cow's milk cheese (five hard, five semi soft and 18 soft) from the dairy collected in 2016 and 2017	*L. monocytogenes* was detected in four samples of soft cheese all at end of production, two at <20 cfu/g, one at 2.3 × 10^3^ cfu/g and one at 1.6 × 10^4^ cfu/g: all isolates were shown to be indistinguishable from that recovered from the clinical samples from the case. Satisfactory results were obtained for all other microbiological parameters: CPS (13 samples), *Salmonella* (12 samples), STEC (five samples) and *E. coli* O157 (nine samples). Unsatisfactory results were obtained for *E. coli* in four soft and one hard cheese sample taken from this producer (levels 2.4 × 10^2^–2.1 × 10^4^ cfu/g): satisfactory *E. coli* levels were detected in six samples. *L. monocytogenes* was not detected in samples with unsatisfactory levels of *E. coli*.
Cheese contamination incident (PHE, unpublished)	Contamination with CPS in 2013	Hard goat's milk cheese which were condemned and did not enter the human food chain.	44 samples of a hard goat's milk cheese were collected over a 2-week period from two separate batches (at the end of production) and produced in the same dairy	*S. aureus* was detected in 40: seven at borderline levels of <10^4^ cfu/g and the remaining 33 at unsatisfactory levels of between 10^4^ and 10^7^ cfu/g. Isolates of *S. aureus* were all shown to contain genes encoding the staphylococcal enterotoxin type C: samples of cheese were tested for staphylococcal enterotoxins which were not detected. Thirty of the samples were tested for other microbiological parameters: *Salmonella* and *Listeria* were not detected in any sample, while five had unsatisfactory levels of *E. coli*, all at <10^3^ cfu/g.

ACCs, aerobic colony counts; CPS, coagulase positive staphylococci; MLST, multilocus sequence type; SNP, single-nucleotide polymorphism type; ST, sequence type; STEC, Shiga toxin-producing *E. coli*.

### Microbiological examination

A 10^−1^ homogenate of each sample was prepared in either maximum recovery diluent, dipotassium hydrogen phosphate buffer or buffered peptone water according to ISO 6887-1:1999 (International Organisation for Standardisation, 1999) and tested using standardised methods (Table 3). All presence/absence tests were performed on single 25 g/ml samples. Overall, of the 2529 samples tested, 7% were tested in Birmingham FW&E laboratory, 27% in London, 44% in Porton, 3% in Preston and 19% in the York laboratory.

**Table 3.** Test methods used for the various microbiological parameters

Microbiological parameters	Test methods
Isolation of *Campylobacter* spp.	ISO 10272-1:2017
Enumeration of CPS, including *S. aureus*	BS EN ISO 6888-1:1999
Isolation of *E. coli* O157	BN EN ISO 16654:2001
Detection of presumptive STEC (*stx* genes) and isolation of STEC	CEN/ISO TS 13136
Isolation and enumeration of *Listeria* spp., including *L. monocytogenes*	BS EN ISO 11290-1:2017 and 11290-2:2017
Isolation of *Salmonella* spp.	ISO 6579:2017
Enumeration of ACCs	BS 4833-2:2013
Enumeration of Enterobacteriaceae	Either based on BS EN ISO 21528-2 2004 or using an automated MPN technique [22]
Enumeration of *β*-glucuronidase producing *E. coli*	Based on BS ISO 16649-2:2001 using either a surface spread or a pour plate technique
Enumeration of coliforms	BS ISO 4832:2006

ACC, aerobic colony counts; CPS, coagulase positive staphylococci; STEC, Shiga toxin-producing *E. coli*.

Microbiological results were interpreted using: Commission Regulation (EC) No. 2073/2005 [13], the Food Hygiene (England) Regulations 2013 [23] and the HPA guidelines for assessing the microbiological safety of ready-to-eat foods placed on the market [14] (Table 4). Data on the detection of STEC *stx* genes (in the absence of the isolation of STEC organisms) were interpreted as satisfactory but is also included in this analysis.

**Table 4.** Criteria for the interpretation of microbiology results

	Satisfactory	Borderline	Unsatisfactory	Unsatisfactory: potentially injurious to health
Bacterial pathogens				
*Campylobacter* in 25 g^a^	Not detected	N/A	N/A	Detected
CPS/g^a^	<20	20 to ⩽10^4^	N/A	⩾10^4^
*E. coli* O157, or any STEC in 25 g	Not detected	N/A	N/A	Detected
*L. monocytogenes*/g^a^	<20	20 to ⩽100	N/A	>100^b^
*Salmonella* in 25 g^a^	Not detected	N/A	N/A	Detected
Indicator organisms				
ACC^c^	<2 × 10^4^	N/A	⩾2 × 10^4^	N/A
Enterobacteriaceae	<10^2^	10^2^ to ⩽10^4^	>10^4^	N/A
*E. coli*/g^a^	<20	20 to ⩽100	>100	N/A
*Coliforms*	<100	N/A	⩾100	N/A
*Listeria* species (not *L. monocytogenes*)/g^a^	<20	20 to <100	⩾100	N/A

N/A, not applicable; STEC, Shiga toxin-producing *E. coli*; CPS, coagulase positive staphylococci.

Results for milk will be in 25 ml and cfu/ml.

a HPA, 2009 [23].

b European Commission, 2005 [13].

c Food Safety and Hygiene (England) Regulations 2013, applicable to raw drinking milk only.

Characterisation of isolates was performed in GBRU using a variety of methods [25–28]. Results were compared with isolates from clinical cases as part of national surveillance. Descriptions of incidents were derived from local investigations.

Descriptive and statistical analysis of the data was undertaken using Excel 2010 (Microsoft, Redmond, Washington). Relative proportions were compared using the Fisher's exact test (GraphPad Software, San Diego, California). A probability value of less than 5% was defined as significant.

## Results and discussion

Amongst all 2529 samples tested, 69% were classified as of satisfactory microbiological quality, 10% were borderline, 16% were unsatisfactory and 5% were unsatisfactory and potentially injurious to health due to the presence of pathogens (Table 5). As previously reported [6], results of statutory hygiene indicator tests for raw drinking milk do not correlate well with the presence of pathogens (see text later). Results of microbiological testing of samples collected during the investigation of incidents and outbreaks of foodborne illness (cow's drinking milk and cheese only) in Table 2 showed a higher overall proportion interpreted as unsatisfactory and unsatisfactory/potentially injurious to health: 44% as compared to 20% for those taken for routine monitoring (Table 5).

**Table 5.** Microbiological quality of samples of raw milk, and dairy products (cream, ice-cream, butter, kefir and cheese) made from unpasteurised milk which were collected in England during 2013–2019

	Number of samples				
	Total tested	Satisfactory	Borderline	Unsatisfactory	Unsatisfactory: potentially injurious to health
All samples	2529	1748 (69%)	249 (10%)	416 (16%)	116 (5%)
Raw milk for drinking					
Outbreaks	56	12 (21%)	17 (30%)	19 (34%)	8 (14%)
Milk, routine monitoring					
Cow's milk (2017–2019)	663	429 (64%)	70 (11%)	140 (21%)	24 (4%)
Goat's milk	534	320 (60%)	81 (15%)	131 (25%)	2 (0.4%)
Sheep's milk	15	12 (80%)	3 (20%)	0	0
Other species' milk	35	26 (74%)	0	9 (26%)	0
Dairy products made from unpasteurised milk					
Cream	100	82 (82%)	11 (11%)	7 (7%)	0
Ice-cream	2	2 (100%)	–	–	–
Butter	37	19 (51%)	8 (22%)	10 (27%)	0
Kefir	24	15 (63%)	0	7 (29%)	2 (7%)
Cheese					
Cheese, incidents and outbreaks	79	41 (52%)	5 (6%)	0	33 (42%)
Cheese, routine monitoring					
Cow's milk	734	589 (80%)	42 (6%)	78 (11%)	25 (3%)
Goat's milk	134	99 (74%)	11 (8%)	9 (7%)	15 (10%)
Sheep's milk	94	87 (92%)	0	5 (5%)	2 (3%)
Milk from other species	22	15 (68%)	1 (5%)	1 (5%)	5 (22%)

Results are presented from routine monitoring which was either performed for the purpose of evaluating the hygiene of foods to support their routine food inspection process (in close collaboration with regulatory authorities) or directly for food manufacturers to support the validation of their food hygiene management systems. For all types of products collected for routine monitoring, similar results to those reported here (Tables 5–8) were found in previous studies in England of raw milk and unpasteurised dairy products [6–10]. Although comparisons between studies should be interpreted with some caution in that sampling may be carried out for different purposes and not strictly co-ordinated within a rigid study design (e.g. based on market share). However, these results show many similarities and identify the same trends as those generated using more formal study designs [24], and we are increasingly utilising this type of data and recognising its risk-based value to provide useful microbiological information from routinely collected food data [6, 15].

**Table 6.** Microbiological results from routine monitoring of raw drinking milk

	ACC	Coliforms	*E. coli*	*Campylobacter*	*L. monocytogenes*	*Listeria* species	CPS	*Salmonella*	*E. coli* O157	STEC
Cow's milk samples (2017–19)										
Total tested	660	654	8	635	642	642	641	622	58	304
Satisfactory	548	557	5	617	637	642	551	619	0	301
Borderline	0	0	2	NA	4	0	90	NA	NA	NA
Unsatisfactory^a^	112	97	1	18	1	0	0	3	0	3^b^
Goat's milk samples (2013–19)										
Total tested	516	385	29	459	472	472	515	464	18	18
Satisfactory	410	399	27	459	472	471	432	464	18	18
Borderline	1	1	1	NA	0	1	81	NA	NA	NA
Unsatisfactory^a^	105	85	1	0	0	0	2	0	0	1
Sheep's milk samples (2013–19)										
Total tested	15	12	1	12	15	15	15	12	1	1
Satisfactory	15	0	1	12	15	15	12	12	1	1
Borderline	0	0	0	NA	0	0	3	NA	NA	NA
Unsatisfactory^a^	0	0	0	0	0	0	0	0	0	0
Milk from other species (2013–19)										
Total tested	35	35	1	32	32	32	33	33	1	0
Satisfactory	31	29	1	0	0	0	31	33	1	–
Borderline	0	0	0	NA	0	0	2	NA	NA	–
Unsatisfactory^a^	4	6	0	0	0	0	0	0	0	–

NA, not applicable; STEC, Shiga toxin-producing *E. coli*; CPS, coagulase positive staphylococci.

a Includes unsatisfactory: potentially injurious to health.

b STEC detected but not isolated from a further two samples.

**Table 7.** Results from routine monitoring of cream, ice-cream, butter, kefir and yoghurt prepared from unpasteurised milk

	Enterobacteriaceae	*E. coli*	*Campylobacter*	*L. monocytogenes*	*Listeria* species	CPS	*Salmonella*
Cream (*n* = 100)							
Total tested	1	97	77	97	97	14	99
Satisfactory	1	74	77	96	96	14	99
Borderline	0	15	NA	1	1	0	NA
Unsatisfactory^a^	0	8	0	0	0	0	0
Ice-cream (*n* = 2)							
Satisfactory	2	2	–	–	–	2	–
Butter (*n* = 37)							
Total tested	25	37	1	37	37	6	36
Satisfactory	19	20	1	37	37	6	36
Borderline	0	8	NA	0	0	0	NA
Unsatisfactory^a^	6	9	0	0	0	0	0
Kefir (*n* = 24)							
Total tested	11	10	24	24	24	23	24
Satisfactory	9	10	24	24	24	21	24
Borderline	0	0	NA	0	0	0	NA
Unsatisfactory^a^	2	0	0	0	0	2	0

NA, not applicable; CPS, coagulase positive staphylococci.

a Includes unsatisfactory: potentially injurious to health.

**Table 8.** Microbiological results from routine monitoring of cheese prepared from unpasteurised milk

	Enterobacteriaceae	*E. coli*	*L monocytogenes*	*Listeria* species	CPS	*Salmonella*	*E. coli* O157	STEC
Total tested	21	787	942	942	903	820	571	142
Satisfactory	13	522	909	929	764	819	570	139^a^
Borderline	3	73	8	1	120	NA	NA	NA
Unsatisfactory^b^	5	192	25	12	19	1	1	3

NA, not applicable; STEC, Shiga toxin-producing *E. coli*; CPS, coagulase positive staphylococci.

a STEC detected but not isolate from a further 10 samples.

b Includes unsatisfactory: potentially injurious to health.

The application of microbiological criteria for the interpretation of results can be problematic with this group of products, with no single guidance document or statutory instrument. In this report, we interpreted the microbiological quality of ready-to-eat foods using legislative criteria [13, 23] and the HPA Guidelines [14]. The HPA guidelines were designed to be applied to ready-to-eat foods placed on the market and should therefore be used with some caution for products collected during production, as is the case here. However, we consider that the use of the HPA Guidelines is appropriate here since only end products were included, and not those during manufacture or food ingredients. Furthermore, the legislative requirements for raw cow's drinking milk require that the point of sale is the same as the point of production. Other dairy products (particularly butter and cream but sometimes cheese) are produced by the same businesses as selling raw milk for drinking (Table 1). Finally, for all types of unpasteurised dairy products, the point of sale can be directly from the manufacturer (including through postal or internet sales), as well as through farm shops which can be co-located with the point of production.

### Raw milk for drinking

The FSA advice recommends business owners to test raw bovine milk for the following: indicator bacteria (*E. coli*, *Listeria* spp., ACCs, coliforms) and pathogenic bacteria (*Salmonella*, STEC, *Campylobacter*, CPS and *L. monocytogenes* [6]). For raw cow's drinking milk, results reported here for samples collected between 2017 and 2019 were similar to those previously reported for 2014–2016 and these two studies provide further baseline data and interpretation for subsequent monitoring of raw cow's drinking milk. There is no evidence to support an improvement in microbiological quality of raw cow's milk for drinking despite the efforts by the Food Standards Agency [1].

For raw milks for drinking which were collected for routine monitoring (Tables 5 and 6), cow's milk were generally of poorer microbiological quality than goat's milk or sheep's milk, both for the presence of indicators as well as for pathogens. Amongst the raw cow's drinking milk tested for routine monitoring purposes, results from 24 samples (4%) were interpreted as unsatisfactory: potentially injurious because of the presence and levels of pathogens (Table 5), no illness were detected as linked to consumption of these products: *Campylobacter* spp. were isolated from 18 of the cow's milk samples (Table 6), 13 of which came from only three producers: results for other parameters were all satisfactory from 13 of the samples, and in remaining five, *Salmonella enterica* serovar Mbandaka was isolated from one, unsatisfactory levels of coliforms were detected in three, and unsatisfactory ACCs were detected in the final sample. In one cow's milk sample there was an unsatisfactory level of *L. monocytogenes* detected (6.8 × 10^2^ cfu/ml): levels of coliforms and ACC were also unsatisfactory for this sample. *Salmonella* was detected in three samples: *S.* Mbandaka was recovered in the presence of *Campylobacter* (see above) and in the remaining two, *S. enterica* serovar Dublin was isolated. Both the samples where *S*. Dublin was detected were collected on different occasions from the same dairy. The levels of coliforms detected were unsatisfactory for both samples while all other microbiological parameters were satisfactory. In the remaining three cow's milk samples categorised as unsatisfactory/potentially injurious to health, STEC was isolated. Two of the isolates both came from different samples collected from the same farm and were both identified as STEC O113:H4, ST10 (*stx*2d; *eae-*negative), the final isolate was identified as STEC O15:H16; ST: 325 (*stx*2g; *eae-*negative); all other microbiological parameters were satisfactory for all three samples.

Two of the goat's milks and none of the milks from sheep or other animals were categorised as unsatisfactory and potentially injurious to health. The unsatisfactory goat's milk samples had high levels of CPS, together with unsatisfactory levels of ACCs and coliforms: both samples came from the same farm and were collected in the same year.

### Cream, ice-cream, butter and kefir

Cream, butter and kefir made from unpasteurised milk are niche products and unlike cheese, are manufactured at the same locations as raw milks (Table 1). Overall, results of microbiological testing classified the majority of the samples of cream, ice-cream, butter and kefir as satisfactory (Table 5). There were no results interpreted as unsatisfactory/potentially injurious to health except for two samples of kefir (Table 7) due to the presence of CPS (10^4^ cfu/g): one was prepared from cow's milk and the other from goat's milk.

There are some difficulties in interpreting the results of hygiene indicators, particularly Enterobacteriaceae, in kefir. Since the microbiota of the kefir grains is usually uncharacterised, these results may be generated by the starter culture and further work on this food type will help to determine an appropriate test profile and interpretation.

### Cheese

Amongst all the 984 cheeses tested as part of routine monitoring, 80% were of satisfactory microbiological quality, 5% were borderline, 10% were unsatisfactory and 5% unsatisfactory/potentially injurious to health. Goat milk cheeses were of poorer microbiological quality than those prepared from milk of other species (Table 5). The 47 cheese were categorised as unsatisfactory potentially injurious to health because of high levels of *L. monocytogenes* or CPS, or the isolation of *Salmonella*, *E. coli* O157 or STEC (Table 8) and a summary of the results from these samples is shown in Table 9. Apart from two possible cases of salmonellosis with indistinguishable *Salmonella* Newport isolated from a hard cow's milk cheese, analysis of national surveillance databases did not provide any other evidence for disease associated with the consumption of these products, or any other of the cheeses sampled here. The samples of cow's, goat's or and sheep milk cheeses collected at the point of production and categorised as unsatisfactory potentially injurious to health were collected from 15 (15%) of the total of 98 dairies sampled. Amongst 41 samples of goat's milk cheese from a single dairy, unsatisfactory levels pathogens were detected in 10 samples: three with unsatisfactory levels of *L. monocytogenes*, five with unsatisfactory levels of CPS and two with unsatisfactory levels of both *L. monocytogenes* and CPS. Although these results may reflect bias from resampling, the decision to sample will have been taken on the recognition of risk within this manufacturer's premises. Therefore within the data presented here, adverse microbiological results were more common in a subgroup of manufacturers where efforts to improve hygiene should be concentrated.

**Table 9.** Summary of microbiological results from testing 47 cheese samples where an interpretation of unsatisfactory potentially injurious to health was obtained

Types of cheese products made from unpasteurised milk (sampling period)	Number of samples	Hazards
Cow's milk cheeses, *n* = 25	13	*L. monocytogenes* was present at >10^2^ cfu/g in all samples, >10^3^ cfu/g in seven and >10^4^ cfu/g in three. The samples were collected from four dairies at the point of production, seven hard cheeses were of a single variety from one dairy and four blue cheeses of the same type from a second producer: the remaining two samples were a hard and semi soft varieties. All other microbiological parameters were satisfactory except for one sample with unsatisfactory ACC levels.
	9	CPS was detected at >10^4^ cfu/g in nine samples which were collected from four dairies at the point of production. Seven samples were of two varieties of hard cheese from two different manufacturers: of the two remaining samples, one was a soft cheese and one could not be classified. All microbiological parameters were satisfactory in seven of the nine samples, unsatisfactory levels of *E. coli* were detected in two.
	1	*S. enterica* serovar Newport ST45 was detected in one sample of a hard cheese collected at the point of production. There were satisfactory results for all other parameters. Analysis of the national database detected two patients infected by a strain of *S.* Newport that was indistinguishable from the isolate recovered from the cheese. The clinical isolates were obtained in the same year as the isolation from cheese and the patient's samples were tested within the same region of the country as the dairy which produced the cheese. No further investigations were recorded.
	2	STEC was isolated from two cheese sample: the organisms were O2:H25, *stx* 2a, *eae*-negative and O2:H27, *stx*2a, *eae*-negative. Both samples were collected from different dairies at production. The types of cheeses could not be classified and all other microbiological parameters were satisfactory.
Goat's milk cheeses, *n* *=* 15	*5*	*L. monocytogenes* was detected at >10^2^ cfu/g in all samples (>10^5^ cfu/g in two samples) which were collected from the same manufacturer at the point of production. Two samples were soft cheeses and the remaining three could not be classified. All parameters were satisfactory in two of the samples, unsatisfactory levels of *E. coli* were detected in one sample and unsatisfactory levels of both *E. coli* and CPS were detected in two samples.
	10	CPS was detected at >10^4^ cfu/g in all samples which were collected from three dairies at the point of production: seven were from a single dairy which was the same as that above where unsatisfactory levels of *L. monocytogenes* were detected. Seven of the cheeses were soft, the remaining three could not be classified. In eight of the samples, all parameters were satisfactory, unsatisfactory levels of both *E. coli* and *L. monocytogenes* were detected in two samples (see above).
	1	STEC O157:H7 (PT 21/18; CC11; *stx*2a *stx*2c; *eae*-positive) was isolated from a mould ripened soft cheese collected at the point of production. All other microbiological parameters were satisfactory except for unsatisfactory levels of *E. coli*.
	1	STEC was isolated from one hard cheese sample: the organism was O6:H10; *stx*1c; *eae*-negative. The sample was collected at the point of production and all other microbiological parameters were satisfactory.
Sheep's milk cheeses, *n* = 2	2	*L. monocytogenes* was detected at >1000 cfu/g from both samples, one collected from production (the cheese type could not be classified), and the second was a fresh cheese collected at retail. All other microbiological parameters were satisfactory.
Cheese prepared from milk of other species, *n* = 5	5	*L. monocytogenes* was detected at >100 cfu/g in all samples (>10^3^ cfu/g in three) of buffalo cheese collected at retail. All samples were identified as produced by the same manufacturer. All other microbiological parameters were satisfactory.

Based on product descriptions, amongst all the 984 cheeses tested as part of routine monitoring, 34 (3%) were classified as fresh, 355 (36%) as hard, 35 (4%) as semi-hard, 57 (6%) as blue, 108 (11%) as semi-soft and 145 (15%) as soft: the remaining 250 (25%) could not be classified. The proportion of semi-soft and soft cheeses with unsatisfactory or borderline levels of CPS (25/102 (23%) for semi-soft and 34/121 (28%) for soft) was significantly higher than for hard and semi-hard cheeses (54/364 (15%): Fisher's exact test; *P* = 0.025 for semi-soft and *P* = 0.002 for soft cheese). Levels of *E. coli* were determined for 576 cheeses that could be classified into different types (hard, soft, etc.), and the distribution of these two parameters is shown in Table 10. The proportion with unsatisfactory levels of *E. coli* (>10^2^ cfu/g) varied from 6% of the hard cheeses, 27% of the semi-hard, to 33–36% of the blue, semi-soft, soft and fresh. The highest levels of *E. coli* occurred in the semi-hard, blue, semi-soft and soft cheese types and corresponded to the types with the lowest proportion of a satisfactory or borderline interpretation.

**Table 10.** Levels of *E. coli* detected as part of routine monitoring of different categories of cheese prepared from unpasteurised milk

Levels of *E. coli*	Number of samples tested in each cheese type (%)					
	Hard	Fresh	Semi-hard	Blue	Semi-soft	Soft
Satisfactory (<20 cfu/g)	211 (84%)	19 (58%)	25 (71%)	22 (39%)	49 (61%)	63 (50%)
Borderline (20 to <10^2^ cfu/g)	25 (10%)	3 (9%)	1 (3%)	10 (18%)	3 (4%)	18 (14%)
Unsatisfactory (10^2^ to <10^3^ cfu/g)	9 (4%)	7 (21%)	3 (9%)	8 (14%)	10 (13%)	17 (14%)
Unsatisfactory (10^3^ to <10^4^ cfu/g)	6 (2%)	4 (12%)	3 (9%)	5 (9%)	8 (10%)	21 (17%)
Unsatisfactory (10^4^ to <10^5^ cfu/g)	0	0	3 (9%)	7 (12%)	10 (13%)	5 (4%)
Unsatisfactory (>10^5^ cfu/g)	0	0	0	0	0	1 (1%)

It is well recognised that there is a relationship between *E. coli* levels and cheese-type with high levels of *E. coli* being more common in soft compared to hard cheese at the end of production or at retail [17, 29]. To achieve process control during manufacture and provides assurance that food safety management plans are operational, cheese makers routinely monitor the microbiological (as well as the physicochemical) quality of the cheese [17, 29]: an important component of this monitoring utilises testing for levels of generic *E. coli*. The UK Specialist Cheesemakers Association has provided criteria for levels of *E. coli*, with a distinction made between hard cheese (<100 cfu/g considered satisfactory) and soft or semi-soft cheese (<10^4^ cfu/g) (http://www.specialistcheesemakers.co.uk/). The Guidance for raw cheese enforcement from the Scottish Food Enforcement Group (https://www.foodstandards.gov.scot/downloads/Guidance_for_Local_Authorities_–_Cheese_made_from_Unpasteurised_Milk_-_May_2019.pdf) recommended that a target level of <100 cfu/g is achievable for some cheese types, and where this is exceeded, further evidence should be provided to verify food safety. The results presented here show an association between higher levels of *E. coli* and the presence of unsatisfactory levels of CPS (Table 11). Therefore, the data indicate that the application of more lenient *E. coli* criteria for unpasteurised cheeses, or soft cheeses, for example, cannot be justified in terms of public health, and it is more appropriate to apply the criteria set out in the HPA guidelines [14] to all ready-to-eat foods, including those made from unpasteurised milk. Both *E. coli* and CPS are likely to reduce as cheeses mature, although the biological risk associated with staphylococcal enterotoxins will remain even in the absence of viable *S. aureus* [30]. The presence of CPS just after manufacture as well as being an indicator of public health risk on its own, may also be a useful indicator of STEC. Furthermore, this should prompt investigation of the use of *E. coli* as an indicator for other raw foods such as raw milk rather than the use of coliforms and ACC.

**Table 11.** Unsatisfactory detection of pathogens at different *E. coli* levels detected as part of routine monitoring of cheese prepared from unpasteurised milk

Levels of *E. coli*			Numbers of samples where pathogen was detected at unsatisfactory, potentially hazardous levels/total number of samples tested (%)			
	*L. monocytogenes*		CPS		STEC isolated	*stx* gene detected
	Borderline	Unsatisfactory	Borderline	Unsatisfactory	Unsatisfactory	
Satisfactory (<20 cfu/g)	4/502 (0.8%)	6/502 (1.2%)	46/513 (9.0%)	19/513 (3.7%)	2/2	2/69 (2.9%)
Borderline (20 to <10^2^ cfu/g)	0/71	0/71	9/70 (12.8%)	9/70 (13%)	–	0/8
Unsatisfactory (10^2^ to <10^3^ cfu/g)	0/72	0/72	19/72 (26.3%)	12/72 (16.6%)	0/3	3/11 (13.6%)
Unsatisfactory (10^3^ to <10^4^ cfu/g)	1/69 (0.14%)	4/69 (5.8%)	15/70 (12.4%)	7/70 (10%)	1/6	6/18 (33.3%)
Unsatisfactory (10^4^ to <10^5^ cfu/g)	1/31 (0.32%)	0/31	3/26 (11.5%)	3/26 (7.7%)	0/2	2/14 (14.3%)
Unsatisfactory (>10^5^ cfu/g)	0/11	0/11	0/11	0/11	–	0/11

STEC, Shiga toxin-producing *E. coli*; *stx, shiga toxin gene*; CPS, coagulase positive staphylococci.

There have been major advances in understanding microbiological hazards in dairy products over the past decades, particularly in relation to STEC [31, 32]. Not only is STEC O157 recognised as a major hazard, but this also extends to other STEC serogroups. The methods to detect and isolate STEC from food matrices are technically demanding and we report here on routine monitoring results obtained from testing 142 cheese prepared unpasteurised milk of which STEC was isolated from four samples: one was STEC O157, and the remaining three were either STEC O2 or O6 (Table 9). Although much effort has been directed towards controlling STEC O157, the isolation of any STEC is unsatisfactory/potentially injurious to health and this is consistent with advice from the Food and Agriculture Organization of the United Nations [18] stated: ‘It is not prudent to regard any STEC strain as being non-pathogenic or not posing a health risk, as all STEC strains probably have the potential to cause diarrhoea and be of risk, especially to susceptible individuals’. Although this presents difficulties to food regulators (as well as food manufacturers), Food Standards Scotland issued a policy statement in 2019 [18] stating ‘the presence of any STEC in a ready-to-eat food is a potential risk to health, and could cause food poisoning’. For this, amongst other reasons, further surveillance is ongoing on the microbiological quality of unpasteurised milk cheeses, including the presence of STEC.

In the three samples where STEC was isolated, *stx* genes were initially detected by polymerase chain reaction. There were a further 10 cheese samples where *stx* genes were detected but STEC was not isolated. The relationship between different levels of *E. coli* and the occurrence of unsatisfactory and borderline levels of *L. monocytogenes* and CPS and the isolation of STEC and detection of *stx* genes are shown in Table 11. The proportion of samples with borderline and unsatisfactory levels of *L. monocytogenes* was similar across the different levels of *E. coli* (Spearman's rank correlation, rho = 0.03, *P* = 0.4). However elevated levels of CPS (Spearman's rank correlation, rho = 0.21, *P* = <0.001) and *stx* detection (Spearman's rank correlation, rho = 0.19, *P* = 0.02) was significantly more likely to occur in the presence of higher levels of *E. coli*. The proportions of samples where STEC was isolated was significantly reduced in the presence of higher levels of *E. coli* (Fisher's exact test *P* = 0.04). The significantly reduced isolation of STEC in the presence of higher levels of generic *E. coli* may reflect the greater technical difficulty in isolating STEC in the presence of a greater competing microbiota (especially non-STEC *E. coli*). The significant association between *stx* detection and higher generic *E. coli* is intriguing but we are unclear how to interpret these results. While the most likely reason for non-isolation is the background microbiota, this may also be as a result of free *stx*-containing phage. Recent studies suggest that the cheesemaking process triggers the production of *stx* containing prophages, potentially interfering with the analysis of STEC in the finished products [33]. These studies demonstrated that oxidative (aeration and exposure to oxygen) and salt stress, which are both likely to occur during cheesemaking, had the ability to induce *stx* phages *in vitro*. Additionally, production of *stx* phages was also observed during cheesemaking when milk was inoculated with a strain of STEC O26.

### Summary

We reviewed here the results of microbiological testing of over 2500 raw drinking milk and dairy products made with unpasteurised milk examined in England between 2013 and 2019. Samples were collected as part of incidents as well as part of routine monitoring and were tested for a range of bacterial indicators and pathogens. This review provides baseline data and interpretation for subsequent monitoring of raw cow's drinking milk and reports a similar level of adverse microbiological results to that reported previously for samples tested between 2014 and 2016: there is no evidence to support an improvement in microbiological quality of this product despite the efforts by the Food Standards Agency. While results presented here, and previously, indicate that the statutory hygiene indicator tests for raw drinking milk do not correlate well with the presence of pathogens. Analysis of data from cheese demonstrated an association between increasing levels of indicator *E. coli* with elevated levels of CPS and detection of *stx* genes. The isolation of STEC was significantly associated with lower levels of indicator *E. coli*. These data provide evidence for setting criteria for *E. coli* in cheeses made from unpasteurised milk. This group of products is a concern for public health, and there is a need for continued surveillance and implementation of controls during production and throughout the food chain.

## Data Availability

The datasets used or analysed during the current study are available from the corresponding author on reasonable request.
